# Out-of-Plane Skew Effects on the Cyclic Performance of Column-Tree Steel Moment Connections

**DOI:** 10.3390/ma19071401

**Published:** 2026-03-31

**Authors:** Geon-Woo Kim, Jong-Kook Hong

**Affiliations:** 1 MS Construction Technology Co., Ltd., 77 Yongbong-ro, Gwangju 61186, Republic of Korea; gwkim7210@nate.com; 2 School of Architecture, Sunchon National University, 255 Jungang-ro, Suncheon 57922, Republic of Korea

**Keywords:** column tree, steel moment connection, out-of-plane skew, bolted splice, cyclic loading, finite element analysis

## Abstract

This study investigates the influence of out-of-plane beam skew on the cyclic performance of column-tree steel moment connections. Utilizing validated finite element (FE) models against experimental data, the cyclic responses of various configurations were evaluated under the AISC cyclic loading protocol up to a story drift ratio of 0.05 rad. Skew angles of 0°, 10°, 20°, and 30° were examined across three representative beam depths. The results demonstrate that all configurations satisfy the AISC 341 acceptance criteria for Special Moment Frames (SMFs), maintaining at least 80% of the plastic moment capacity (0.8 *M_p_*) up to a 0.04 rad story drift ratio. However, the introduction of beam skew resulted in a gradual reduction in energy dissipation capacity, with the total dissipated energy decreasing by 2.9–8.9% at a 30° skew. Notably, the inelastic energy component was more sensitive to the skew than the frictional components, exhibiting a maximum reduction of 15.4%. While out-of-plane skew disrupted the symmetry of stress triaxiality and plastic strain at the beam-to-column interface, the overall fracture susceptibility was not significantly exacerbated up to 30°. Furthermore, column twisting remained within a negligible range (below 0.5°), and its impact on global stability was limited. Despite the general stability, a premature bolted splice failure was observed in deep beam configurations at a 30° skew during the 0.05 rad drift cycles. Based on these findings, it is concluded that column-tree connections with an out-of-plane skew up to 30° are viable; however, a design limit of 20° is recommended for deep beam configurations to ensure structural integrity under extreme cyclic demands.

## 1. Introduction

Steel special moment frames (SMFs) are widely utilized as primary lateral-force-resisting systems in seismic regions, owing to their superior ability to develop stable hysteretic behavior and dissipate seismic energy through controlled inelastic deformation. In contemporary practice, particularly in Korea and Japan, column-tree framing has emerged as a preferred construction methodology. As highlighted by Cucuzza et al. [1], modern structural efficiency is increasingly driven by constructability-based design, which emphasizes the standardization of members and connections to mitigate assembly complexity. The column-tree system exemplifies this trend by transitioning critical, high-precision welding to controlled shop environments, thereby restricting field assembly to standardized bolted splices, which significantly enhances overall fabrication quality.

Despite these practical advantages, current seismic design provisions, such as KDS 14 31 60 [2] and ANSI/AISC 341 [3], provide relatively sparse guidance regarding the specific nuances of column-tree connections. While experimental work by Oh [4] confirmed that most column-tree configurations satisfy SMF requirements, the results underscored the fact that global performance is highly sensitive to the mechanical behavior of the field splice. Previous research [5,6] has shown that splice slip often precedes the yielding of primary members, with actual friction coefficients falling significantly below the nominal Class B values. Furthermore, numerical investigations [7] have identified progressive pretension loss following splice slip; for instance, flange-bolt forces have been observed to drop to 20–30% of their initial pretension (*T_o_*) at story drift ratios between 0.015 and 0.03 rad.

**Figure 1:**
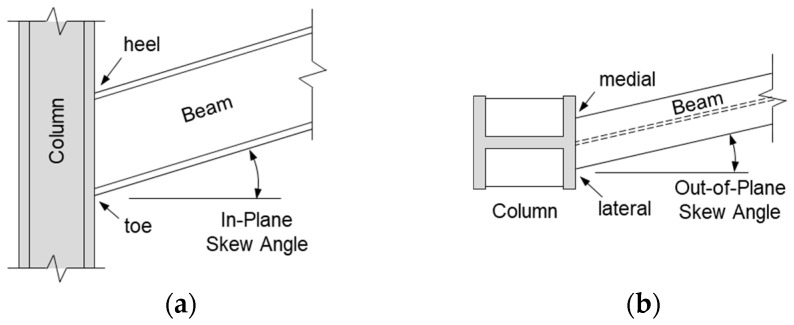
Non-orthogonal beam–column connections: (**a**) in-plane skew (sloped connection); (**b**) out-of-plane skew (skewed connection).

The demand for architectural flexibility in irregular floor plans frequently necessitates non-orthogonal framing, resulting in either in-plane or out-of-plane skew (see Figure 1). Despite its prevalence, design guidance for skewed configurations remains inadequate, resulting in some conservative guidelines recommending their exclusion from the primary seismic system [8]. To date, research has predominantly focused on in-plane skew, with studies [9,10,11] identifying a high risk of brittle “heel weld” failure and suggesting that the skew angle be strictly limited to 10°.

In contrast, research into out-of-plane skews has been largely confined to reduced beam section (RBS) [12,13] or end-plate connections [14], where increased column twisting and elevated bolt demands due to combined bending and torsion were reported. For column-tree systems specifically, the impact of out-of-plane skew remains a critical research gap. Preliminary monotonic studies [15] suggest that such geometry may reduce splice-bolt forces while increasing demand on the welded beam-to-column interface; however, its performance under cyclic loading is not yet fully understood.

Building upon these findings, this study addresses the aforementioned gap by systematically investigating the effects of out-of-plane beam skew on the cyclic responses of column-tree moment connections. Utilizing three experimentally validated finite element (FE) models, the research extends the investigation from orthogonal (0°) to substantial skew angles (up to 30°). This analysis specifically aims to:

- Quantify the torsional–flexural coupling that governs hysteretic stability and energy dissipation.
- Establish the correlation between skew angle and splice-bolt force retention under cyclic reversals.
- Benchmark the susceptibility of welded beam-to-column regions to fracture under non-orthogonal loading conditions.

Ultimately, these results provide a technical foundation for developing design limits that ensure the structural integrity of non-orthogonal column-tree systems in moderate-to-high seismic regions.

## 2. Connection Configuration

**Figure 2:**
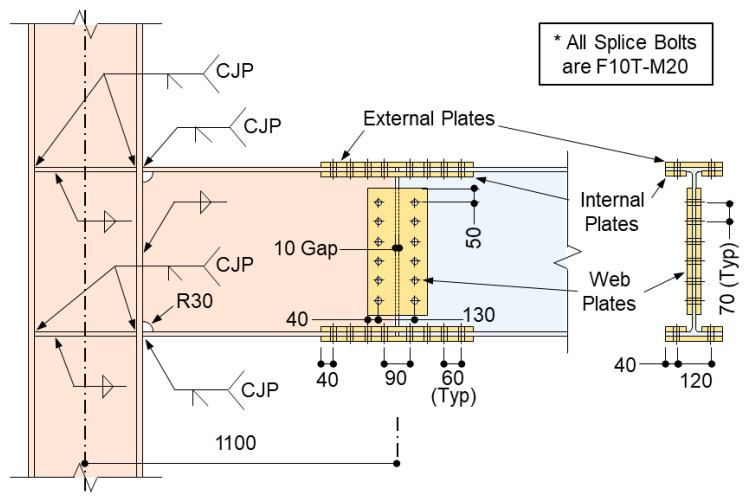
Column-tree connection configuration.

The geometric configurations analyzed in this study were based on the column-tree connection details experimentally validated by Oh [4]. As illustrated in Figure 2, the setup consists of a single-sided moment connection comprising a column and a short stub beam. The stub-beam flanges are joined to the column flange using complete joint penetration (CJP) groove welds, while the web is connected via fillet welds. A field-bolted splice is positioned 1100 mm from the column centerline, incorporating a 10 mm gap between the stub beam and the main beam segment. The splice utilizes 20 mm diameter high-strength bolts (F10T-M20), designed as a slip-critical joint. To facilitate double-shear behavior, the splice assembly includes both external and internal flange plates, along with web plates.

The column is fabricated from SM355 steel (nominal yield strength, *F_y_* = 355 MPa), while the beam and splice plates consist of SM275 steel (*F_y_* = 275 MPa). Three representative beam sections—H-400 × 200 × 8 × 13, H-500 × 200 × 10 × 16, and H-600 × 200 × 11 × 17—were analyzed in conjunction with an H-400 × 400 × 13 × 21 column.

The splice components for each model are summarized in Table 1. While the flange splice details for Models 5 and 6 are identical in terms of bolt arrangement, they differ in plate thickness. Additionally, the web-splice plate thickness and bolt quantity were increased proportionally with the beam depth to ensure adequate load transfer across the different configurations.

## 3. Finite Element Modeling and Verification

### 3.1. Numerical Model and Element Types

**Figure 3:**
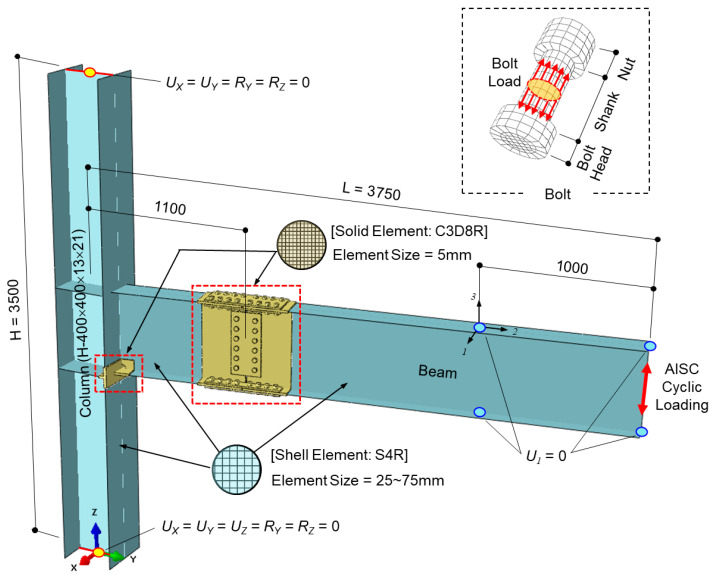
Finite element model and boundary conditions of the column-tree connection.

The finite element analysis (FEA) was performed using ABAQUS [16] to evaluate three representative models (Models 4, 5, and 6), as depicted in Figure 3. The overall configuration, boundary conditions, and loading protocols were designed to replicate the cyclic testing conditions reported by Oh [4]. Each model consisted of a half-span beam (*L* = 3750 mm) connected to the mid-height of a full-story column (*H* = 3500 mm).

Regarding the boundary conditions, the column base and top were restrained to simulate hinged and roller supports, respectively. To prevent premature lateral-torsional buckling, out-of-plane displacements (*U*_1_) of the beam flanges were restrained at the loading point (beam end) and at lateral brace locations 1000 mm from the beam end. To maintain a stable vertical load path and focus the investigation on the connection’s hysteretic response, lateral-torsional buckling (LTB) was suppressed using idealized lateral restraints. While skewed beams in actual floor diaphragms may experience additional torsional demands or sway due to non-orthogonal bracing, these idealized constraints ensure numerical stability and a controlled environment consistent with standard laboratory testing protocols. This allows for a clear quantification of the skew-induced effects on the joint itself, independent of global member instabilities.

The M20 high-strength bolts were simplified as cylindrical bodies representing the bolt head and nut; washers and threads were not explicitly modeled to optimize computational efficiency. Hexagonal bolt heads and threads often cause chattering or convergence issues in nonlinear contact algorithms due to their sharp corners. In contrast, cylindrical heads with an equivalent bearing area and non-threaded shanks are standard FEA practices used to overcome analytical instabilities. While this simplification may slightly overestimate the initial axial stiffness by omitting the localized compliance of thread engagement and washers, it remains a robust approach for capturing the macro-mechanical relaxation of the bolt shank. This relaxation, driven by global splice slip and cyclic reversals, is the primary governor of joint integrity in large-scale assemblies. This modeling strategy is consistent with the systematic approach recommended by VDI 2230 [17] and validated by Grizejda [18], ensuring that the fundamental load transfer mechanisms are preserved while maintaining computational stability. Bolt holes were modeled 2 mm larger than the bolt diameter to account for standard clearance, in accordance with KDS 41 [5].

To balance computational accuracy and efficiency, eight-node linear solid elements with reduced integration (C3D8R) were assigned to the critical regions, including the beam–column interface and the bolted splice connections. The remaining components were discretized using four-node linear shell elements with reduced integration (S4R). The mesh size for solid elements was capped at 5 mm in critical zones to ensure high-fidelity results, while the shell element sizes ranged from 25 mm near the connection area to 75 mm at the distal ends of the beam and column.

### 3.2. Contact Interactions and Constraints

To simulate the complex behavior of slip-critical joints, contact interactions were defined across all mating surfaces, including beam flanges–flange splice plates, beam web–web splice plates, splice plates–bolt heads/nuts, and bolt shanks–bolt holes. Establishing appropriate contact properties was essential for capturing the nonlinear response of the column-tree connection accurately. For these interfaces, tangential behavior was modeled using the Coulomb friction law with the penalty method, while normal behavior was governed by the “hard contact” relationship.

The friction coefficient at the faying surfaces under cyclic loading varies significantly in the existing literature, with reported values ranging from 0.08–0.38 [4], 0.265 [7], and 0.20 [19]. These values are considerably lower than the code-specified slip coefficient (=0.50 for Class B surfaces), which is typically derived from monotonic loading conditions. This discrepancy arises because fretting wear and surface polishing during cyclic reversals lead to a degradation of the friction coefficient. In this study, a friction coefficient of 0.20 was adopted following the recommendation of Matthews and Nunez [19], ensuring that the numerical results aligned closely with the experimental data.

**Figure 4:**
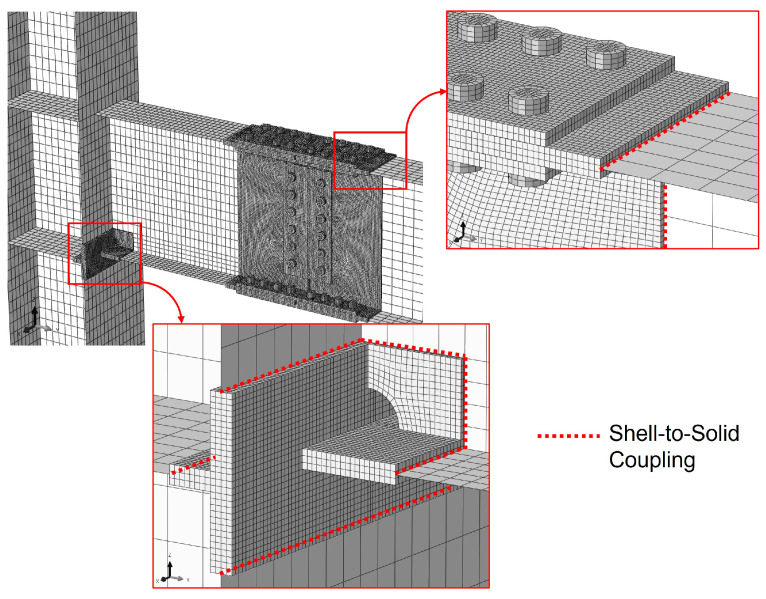
Shell-to-solid constraint.

As the FE models utilized a combination of shell and solid elements, it was necessary to address the incompatibility in degrees of freedom (DOFs) at their interfaces. A shell-to-solid coupling constraint was employed to ensure a kinematically consistent transition by tying the displacement and rotation of each shell node to the average displacement and rotation of the adjacent solid surface [16]. An element-based solid surface was selected for this coupling, as illustrated in Figure 4. Regarding the welded joints, such as the beam-to-column and column flange-to-continuity plate connections, weld geometries were not explicitly modeled. Instead, these components shared common nodes at the interfaces, effectively behaving as a monolithic unit.

### 3.3. Material Properties

Mild steel subjected to monotonic loading is typically characterized by strain hardening following the initial yield. However, under cyclic loading, it exhibits complex plastic behavior, including cyclic hardening/softening, material ratcheting, and the Bauschinger effect. To accurately capture these phenomena, the stress–strain relationship under cyclic loading must be represented by isotropic hardening, kinematic hardening, or a sophisticated combination of both.

In this study, a nonlinear combined isotropic/kinematic hardening model was employed. The numerical parameters for this constitutive model were obtained from the established literature for various steel grades [20,21,22]. Specifically, calibrated values derived from cyclic coupon testing for Korean steel grades (SM275 and SM355) [22] were utilized to ensure the reliability of the seismic response.

**Figure 5:**
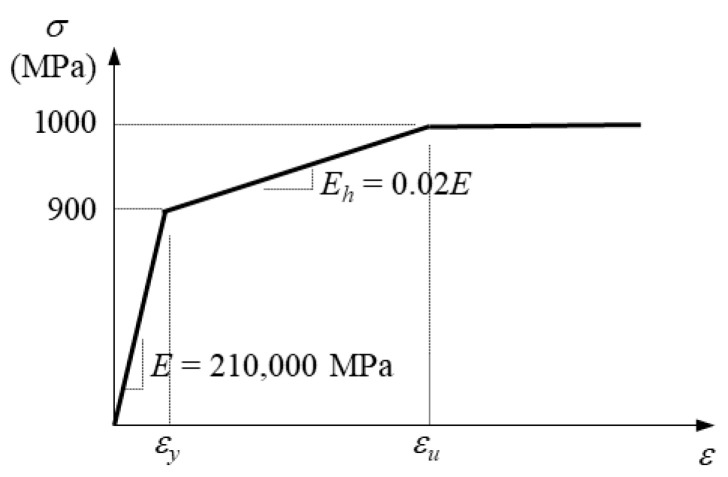
Assumed stress–strain relation for high-strength bolt (F10T).

Regarding the high-strength bolts in the splice, previous experimental observations [4] indicated no distinct deformation or structural failure. Accordingly, the bolts were not expected to undergo significant plastic deformation throughout the cyclic loading protocol. Nevertheless, to maintain analytical robustness, a tri-linear stress–strain relationship—encompassing the elastic range, a strain-hardening transition, and the ultimate plastic region [23]—was assumed for the bolts, as illustrated in Figure 5.

### 3.4. Bolt Pretension and Cyclic Loads

Loading was applied in two sequential stages: bolt pretensioning, followed by the cyclic loading stage. In the initial stage, the minimum bolt pretension of 165 kN, as specified in KDS 41, was gradually applied to the center of each bolt shank using the “bolt load” feature in ABAQUS (see Figure 3). Once the designated pretension was achieved, the bolt length was fixed (using the “fix at current length” option) to maintain the clamping force throughout the subsequent analysis steps.

**Figure 6:**
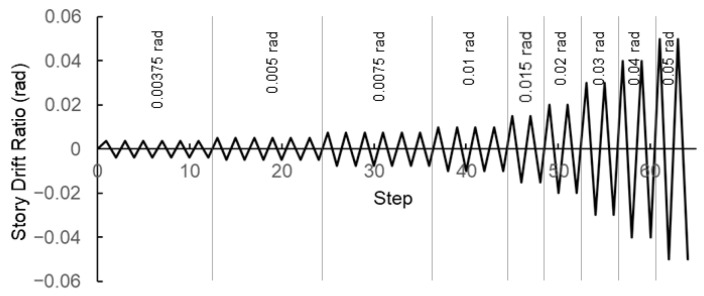
AISC loading protocol.

In the second stage, a displacement-based cyclic loading protocol was applied to the beam end, following the AISC Seismic Provisions (AISC 341), reaching a maximum story drift ratio of 0.05 rad. The specific AISC loading history for beam-to-column moment connections is illustrated in Figure 6.

To ensure numerical convergence and mitigate potential stability issues arising from large displacements and complex contact interactions under cyclic reversals, a dynamic implicit solver incorporating a quasi-static procedure was employed. This approach effectively manages the material and geometric nonlinearities inherent in slip-critical joint behavior, as well as the large-scale deformations of the structural system.

### 3.5. Analysis Model Verifications

**Figure 7:**
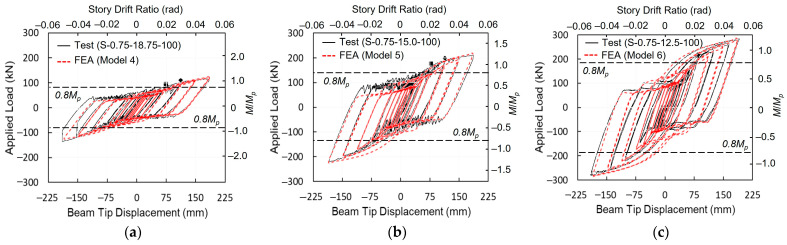
Load versus displacement verification of baseline models: (**a**) Model 4; (**b**) Model 5; and (**c**) Model 6. (Test from [4]).

The load–displacement relationships for each model were compared with the experimental results, as illustrated in Figure 7. The overall hysteretic responses—including the initial stiffness, post-yield stiffness, bolt slip loads, and peak loads at each drift level—demonstrated excellent agreement with the test data.

Table 2 summarizes the performance comparison between the numerical analysis and the experimental results, specifically focusing on initial stiffness, average slip loads during the 0.02 rad cycles, and the maximum load achieved at 0.05 rad. The discrepancies between the analytical and experimental results ranged from −7.1% to +7.5%, which is within an acceptable engineering margin.

Given the inherent experimental uncertainties—such as variations in actual material properties, the precise positioning of bolts within their holes, minor deviations in bolt hole diameters, and the specific boundary conditions imposed by the testing apparatus—the FE analysis results are considered to be well-correlated with the experimental behavior. This high level of correlation validates the proposed modeling approach, providing a reliable foundation for further investigation into skewed configurations.

## 4. Results and Discussion of Out-of-Plane Skew

### 4.1. Parametric Study for Out-of-Plane Skew

Following the successful validation of the three representative baseline models (Models 4, 5, and 6), this study was extended to evaluate the influence of out-of-plane beam skews on the cyclic behavior of column-tree moment connections.

Using the orthogonal beam-to-column configuration as a control group, the out-of-plane skew angle was increased in 10° increments up to a maximum of 30°. Consequently, three skewed configurations were developed for each baseline model, resulting in a total of nine skewed case models. To ensure consistency and comparability across all simulations, each model was constructed and analyzed using the identical modeling methodology and material parameters established in the previous sections.

### 4.2. Cyclic Behavior

**Figure 8:**
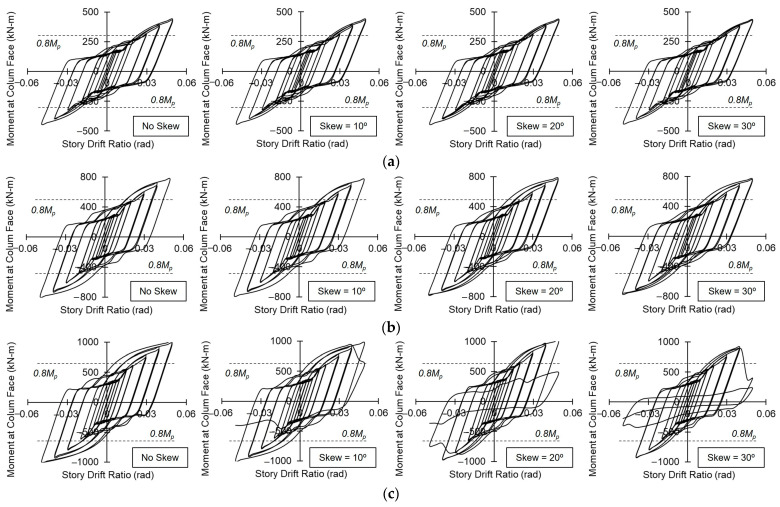
Global response: (**a**) Model 4; (**b**) Model 5; and (**c**) Model 6.

The moment versus story drift ratio hysteresis responses for all analyzed cases are presented in Figure 8. These responses are characterized by an initial bolt slip within the splice, followed by strain hardening due to the inelastic deformation of the beam. Splice bolt slips typically initiate at a story drift ratio between 0.0075 and 0.01 rad, with strain hardening becoming prominent during the 0.01 rad and 0.015 rad drift cycles.

All models successfully completed two full cycles at a story drift ratio of 0.04 rad. The moment-resisting capacity consistently remained above 0.8 *M_p_*, demonstrating excellent ductility and energy dissipation regardless of the skew angle. These results indicate that the out-of-plane skewed connections satisfy the acceptance criteria for Special Moment Frames (SMFs) as specified in the AISC Seismic Provisions (AISC 341). Furthermore, the hysteresis responses revealed no significant deviations between the orthogonal and skewed configurations regarding the bolt slip range, hysteretic energy (area under the curve), or peak moment capacity up to a drift ratio of 0.04 rad.

However, despite satisfying the SMF requirements at 0.04 rad, Model 6, with an out-of-plane skew angle, experienced failure in the bolted splice connection, accompanied by a sudden loss of strength during the 0.05 rad story drift cycles. In these specific skewed configurations of Model 6, excessive bolt hole elongation in the splice plates and shear deformation of the flange bolts were observed at the conclusion of the analysis. This premature splice failure is likely attributable to the relatively thinner flange splice plates used in Model 6 compared to those in Model 5, which reduced the bearing and shear resistance under high-amplitude cyclic demands.

### 4.3. Energy Dissipation

Conventional steel special moment frames (SMFs), primarily consisting of welded beam-to-column connections, dissipate seismic energy through the inelastic deformation of structural components. In contrast, column-tree moment frames incorporate bolted splice connections designed to slip under cyclic loading. The resulting friction contributes significantly to the system’s overall energy dissipation capacity. In this study, both inelastic and frictional energy dissipation components were quantified using the internal energy output options in ABAQUS.

**Figure 9:**
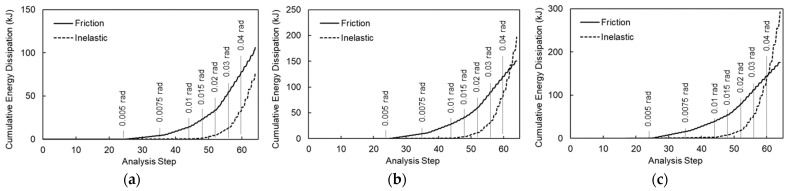
Energy dissipation of baseline models: (**a**) Model 4; (**b**) Model 5; and (**c**) Model 6.

Figure 9 illustrates the cumulative inelastic and frictional energy dissipation throughout the cyclic analysis for the baseline models. Markers indicate the completion of each designated story drift ratio for reference. Frictional energy dissipation within the bolted splice connections initiated at a story drift ratio of 0.0075 rad. This was followed by inelastic energy dissipation due to material nonlinearity, which became prominent between the 0.01 rad and 0.015 rad drift cycles. While frictional dissipation was dominant during smaller story drift cycles, the inelastic component increased rapidly as the drift intensified. Notably, in Model 4, the frictional component remained higher than the inelastic component throughout the entire analysis. However, the inelastic component surpassed the frictional energy during the 0.05 rad cycles in Model 5, and just before the conclusion of the 0.04 rad cycles in Model 6.

**Figure 10:**
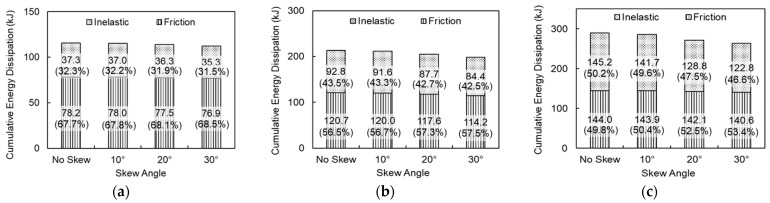
Cumulative energy dissipation at the end of 0.04 rad cycles: (**a**) Model 4; (**b**) Model 5; and (**c**) Model 6.

The relative contributions of each mechanism at the end of the 0.04 rad drift cycle are presented in Figure 10. For Model 4, which features a shallower beam, the frictional energy contribution was consistently more than double that of the inelastic energy. As the beam depth increased, the inelastic contribution accordingly rose; in Model 6, the inelastic and frictional energy components were nearly equal in magnitude.

The introduction of an out-of-plane skew resulted in a gradual decrease in both energy dissipation components across all models. This reduction was most pronounced in configurations with larger skew angles, where the inelastic energy component was more significantly affected than the frictional component. Table 3 summarizes the effects of out-of-plane skews on energy dissipation up to the 0.04 rad story drift ratio. Total energy dissipation decreased as the skew angle increased, with the most significant reduction observed in Model 6. For a 30° skew angle, the total energy dissipation was reduced by 2.86%, 6.98%, and 8.92% for Models 4, 5, and 6, respectively, compared to their orthogonal counterparts. Specifically, the reduction in the inelastic component reached 5.35% in Model 4, 9.05% in Model 5, and 15.43% in Model 6, whereas the reduction in the friction component was limited to 1.66–5.39%.

The higher sensitivity of inelastic energy dissipation to the skew angle, compared to frictional dissipation, is fundamentally linked to the alteration of the plastic hinging mechanism. While frictional work is a localized mechanism governed by contact physics and bolt clearance, material yielding is a global response sensitive to force-path redirection. In skewed configurations, the interaction of bending and torsion induces asymmetric yielding, where stress concentrations at the medial joints (acute flange corners) lead to a less efficient distribution of plastic strains across the section. This reduction in the ‘active’ plastic volume directly accounts for the observed 15.4% decrease in energy dissipation in Model 6, as the section fails to develop a fully symmetric plastic hinge compared to the orthogonal (0°) benchmark.

### 4.4. Beam–Column Connection Fracture Susceptibility

Accurately quantifying the fracture susceptibility of beam-to-column welded connections in finite element analysis remains a challenge, as microscale fracture mechanisms are not explicitly modeled. Instead, fracture susceptibility is typically estimated using response indices [24] based on stress, strain, or a combination thereof. This study considers three specific indices: the Triaxiality Ratio (*TR*), associated with brittle fracture; the Equivalent Plastic Strain Index (*PI*), related to ductile crack initiation; and the Rupture Index (*RI*), which represents the overall susceptibility to fracture. Higher index values indicate a greater propensity for connection failure. These response indices are defined as follows:

(1) $$TR=\frac{\sigma_{m}}{\sigma_{eff}}$$

(2) $$PI=\frac{PEEQ}{\varepsilon_{y}}$$

(3) $$RI=\frac{PI}{exp\left(-1.5\cdot TR\right)}$$

where $\sigma_{m}$ is the hydrostatic stress; $\sigma_{eff}$ is the von-Mises stress; $PEEQ=\sqrt{\frac{2}{3}\varepsilon_{ij}\varepsilon_{ij}}$ is the equivalent plastic strain; $\varepsilon_{ij}$ is the plastic strain component in direction *i* and *j*; and $\varepsilon_{y}$ is the yield strain.

**Figure 11:**
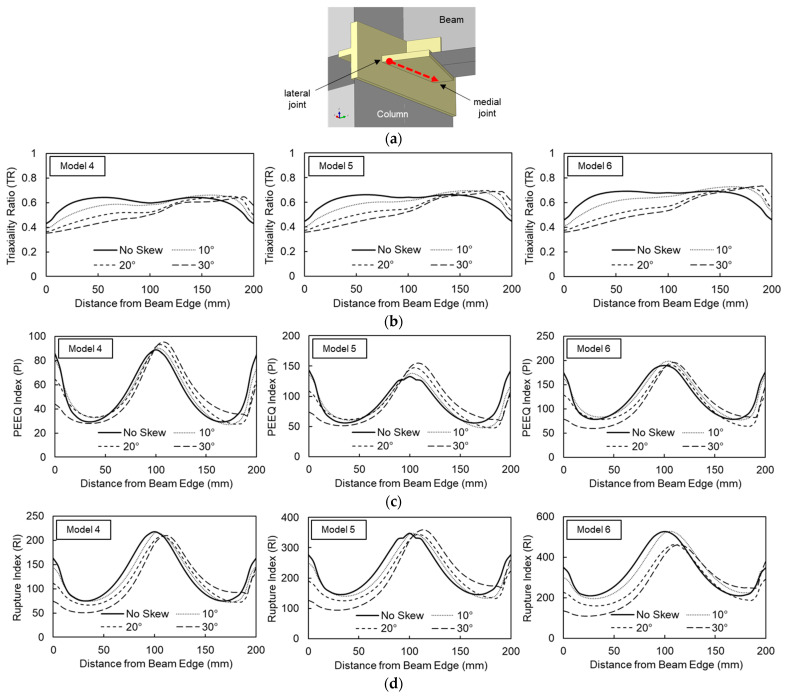
Fracture index at story drift ratio of 0.04 rad: (**a**) Location; (**b**) Triaxiality Ratio (*TR*); (**c**) Plastic Equivalent Strain Index (*PI*); (**d**) Rupture Index (*RI*).

The distribution of these response indices along the bottom flange adjacent to the beam-to-column interface at a story drift ratio of 0.04 rad is presented in Figure 11. For orthogonal (non-skewed) beams, the response indices exhibited a symmetric distribution relative to the beam centerline. This symmetry was disrupted with the introduction of out-of-plane beam skew. As the skew angle increased, *TR* values at the medial joint (where the beam flange intersects the column at an acute angle) increased, while *TR* values at the lateral joint (the obtuse side) decreased. The most significant increase in the *TR* index was observed in Model 6, where the value at the medial joint rose from 0.46 (orthogonal) to 0.73 (30° skew). However, this localized increase was not considered critical, given that the peak *TR* index across the entire flange width of the base model was 0.69.

*PI* indices peaked at the center of the beam flange in the baseline models. With the introduction of out-of-plane skew, the peak location shifted slightly toward the medial joint, accompanied by an increase in magnitude. At a 30° skew angle, the peak *PI* index increased by 22.7%, 8.4%, and 3.4% for Models 4, 5, and 6, respectively, compared to their orthogonal counterparts.

Similarly, the highest *RI* values were recorded at the center of the beam flange in the base models, with the peak location shifting toward the medial joint as skew was introduced. Overall, the *RI* magnitude across the flange width generally decreased with out-of-plane skew, with the exception of Model 5 at a 30° skew, which showed a marginal peak *RI* increase of 3.4%.

In summary, the fracture susceptibility of the welded beam-to-column connections in the column-tree assemblage was not significantly exacerbated by out-of-plane skews up to 30°. This result is consistent with the observed reduction in inelastic energy dissipation in skewed configurations, which alleviates the localized demand on the welded regions.

### 4.5. Bolt Force

**Figure 12:**
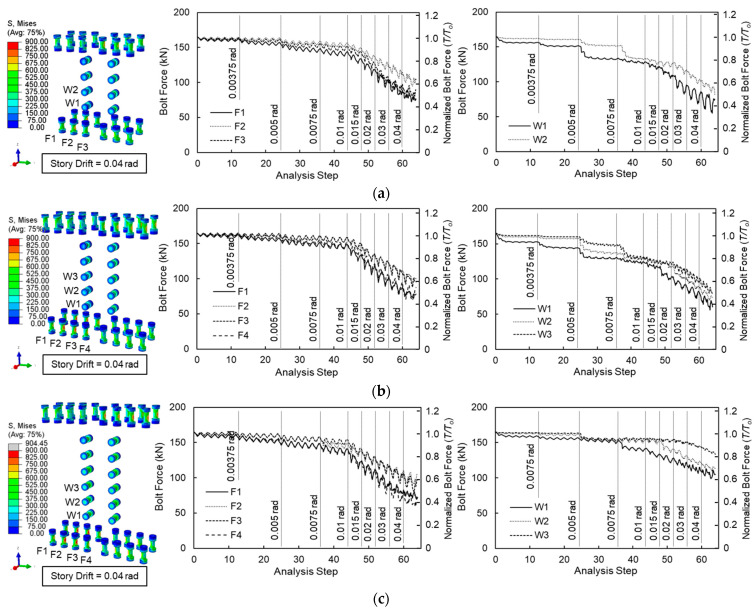
Splice bolt stress and force of baseline models: (**a**) Model 4; (**b**) Model 5; and (**c**) Model 6.

When column-tree moment frames are subjected to cyclic loading, the slip-critical joint splices undergo bolt slippage, which subsequently affects the pretension levels within the bolts. Figure 12 illustrates the von-Mises stress distribution in the splice bolts at a story drift ratio of 0.04 rad, alongside the variation in bolt axial forces throughout the cyclic loading for the baseline models. At 0.04 rad, the von-Mises stress in most bolts remained below the nominal yield strength of F10T (*F_y_* = 900 MPa); however, the peak stress reached 904 MPa in Model 6, indicating localized yielding.

The axial force in the bolts decreased as the story drift ratio increased. In the flange splice bolts, the initial pretension (*T_o_* = 165 kN) gradually declined until a story drift ratio of 0.01 rad, followed by a rapid drop from the 0.015 rad cycles onward. At a story drift ratio of 0.04 rad, the flange bolt forces were reduced to 82.4 kN (=0.50*T_o_*), 80.6 kN (=0.49*T_o_*), and 65.0 kN (=0.39*T_o_*) for Models 4, 5, and 6, respectively.

For the web splice bolts, the axial force began to decrease significantly starting from the 0.02 rad cycles, following a gradual, stepped loss during the lower drift cycles. At 0.04 rad, the minimum web splice bolt forces were recorded as 68.1 kN (=0.41*T_o_*), 72.6 kN (=0.44*T_o_*), 106.3 kN (=0.64*T_o_*) for Models 4, 5, and 6, respectively. It was observed that the rate of initial pretension loss was higher in the flange splice bolts than in the web splice bolts for deeper beam configurations. Conversely, for shallower beams, the web splice bolts were more susceptible to pretension loss.

**Figure 13:**
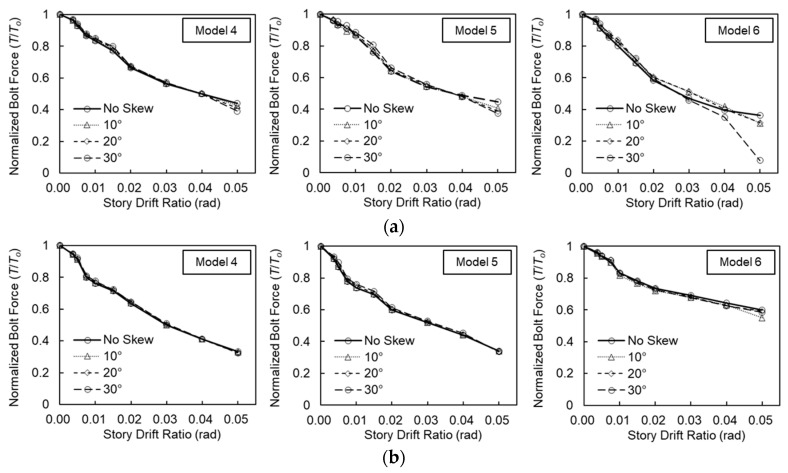
Out-of-plane skew on splice bolt force: (**a**) flange splice bolt; (**b**) web splice bolt.

Figure 13 illustrates the effects of out-of-plane skews on the splice bolt forces. Bolt forces, normalized to the initial pretension (*T_o_*), are plotted against the story drift ratio. Up to a story drift ratio of 0.04 rad, only minor deviations were observed between the orthogonal and skewed configurations within each model. This suggests that the influence of out-of-plane skews on splice bolt pretension in column-tree connections is negligible. The most significant deviation occurred in Model 6 with a 30° skew angle during the 0.05 rad cycles, which is attributed to the eventual failure of the splice connection.

### 4.6. Splice Connection Rotation

**Figure 14:**
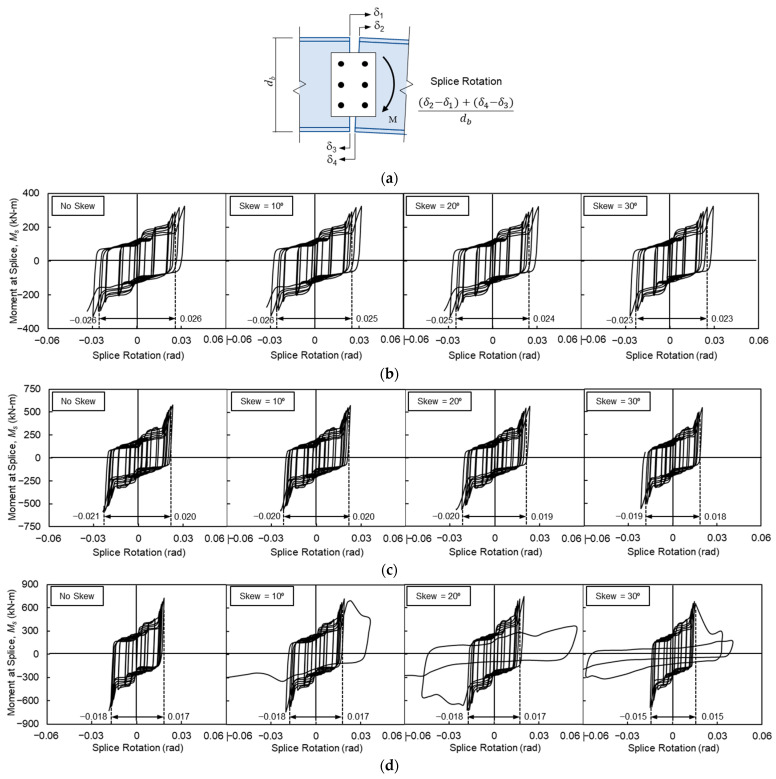
Splice rotation: (**a**) definition of splice rotation; (**b**) Model 4; (**c**) Model 5; and (**d**) Model 6.

The performance of the bolted splice connection was evaluated by examining the relationship between the applied moment and splice rotation, as illustrated in Figure 14. Splice rotation was defined as the relative displacement between the stub beam and the main beam segment, divided by the beam depth. This relative displacement represents the differential movement between the top and bottom flanges within the splice, primarily induced by bolt slippage and the deformation of the splice plates. The moment at the splice, *M_s_*, was calculated as the product of the applied load at the beam end and the distance from the loading point to the centerline of the splice connection. Vertical dashed lines in Figure 14 indicate the extent of splice rotation up to a story drift ratio of 0.04 rad.

The results indicate that splice rotation decreases as beam depth increases. For the baseline (orthogonal) models, the splice rotation ranged from −0.026 to 0.026 rad, −0.021 to 0.020 rad, and −0.018 to 0.017 rad for Models 4, 5, and 6, respectively. These values demonstrate that in the absence of skew, splice rotation contributes significantly to the total deformation, accounting for 65.0%, 52.5%, and 45.0% of the overall story drift ratio for Models 4, 5, and 6, respectively.

The introduction of an out-of-plane skew resulted in a slight reduction in splice rotation compared to the respective baseline models. However, this reduction remained minimal across most configurations, with the notable exception of Model 6 at a 30° skew angle. The significant increase in splice rotation observed for Model 6 in the skewed configuration suggests a loss of structural integrity and subsequent failure of the splice connection during the 0.05 rad drift cycles.

### 4.7. Column Twisting

**Figure 15:**
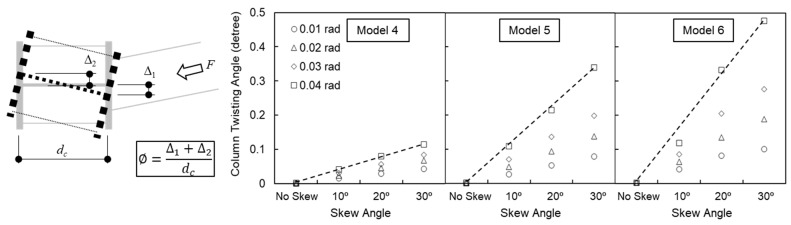
Column twist angle.

Out-of-plane beam skew introduces out-of-plane shear forces at the beam-to-column interface, which inevitably results in column twisting. Excessive twisting is detrimental as it can reduce the overall load-carrying capacity and lateral stability of the structural system. The column twist angle (ϕ), observed in the analytical models up to a story drift ratio of 0.04 rad, is presented in Figure 15. The twist angle was derived from the differential out-of-plane displacements between the column flanges at the level of the beam’s top flange.

Column twisting in the orthogonal baseline models was negligible. However, the twist angle increased almost linearly with the out-of-plane skew angle. At a 30° skew angle and 0.04 rad story drift, the column twist angles were recorded as 0.11°, 0.34°, and 0.48° for Models 4, 5, and 6, respectively. Across all analyzed cases, the magnitude of column twisting remained minimal, suggesting that the out-of-plane skew angles examined in this study do not pose a significant risk to the global stability of the column in this configuration.

**Figure 16:**
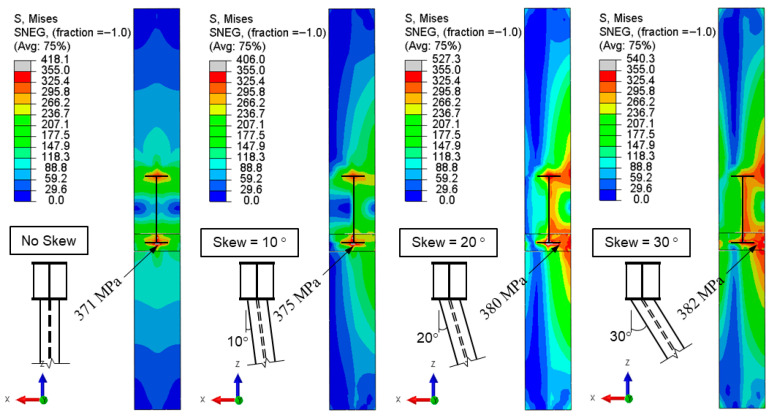
Model 6: von-Mises stress in the column flange at a story drift ratio of 0.04 rad.

Column twisting induces a non-uniform stress distribution, which may increase the local demands on the welded beam-to-column connection. Figure 16 illustrates the von-Mises stress distribution on the column flange for Model 6, comparing the orthogonal and skewed configurations at a story drift ratio of 0.04 rad. In the baseline model, the stress distribution across the column flange was symmetric, with a peak stress of 371 MPa observed at the beam flange centerline.

With the introduction of out-of-plane skew, the symmetric stress distribution was disrupted, leading to stress concentrations in the medial joint region. For a 30° skew angle, the peak stress in this region rose to 382 MPa—a marginal increase compared to the baseline model. These findings suggest that while out-of-plane beam skew alters the localized stress distribution within the column, its overall impact on the structural integrity of the welded beam-to-column connection is minor.

## 5. Conclusions

This study numerically investigated the influence of out-of-plane beam skew on the cyclic performance of column-tree steel moment connections. Based on the evaluation of three representative configurations with skew angles ranging from 0° to 30°, the following conclusions are drawn:

1. Global Cyclic Response: Up to a story drift ratio of 0.04 rad, out-of-plane skew exerted a negligible effect on the initial stiffness, splice slip range, and peak strength. All configurations maintained at least 80% of their plastic moment capacity (0.8 *M_p_*) through the 0.04 rad cycles, successfully satisfying the AISC 341 acceptance criteria for Special Moment Frames (SMFs). However, the deep beam model (Model 6) with a 30° skew exhibited sudden strength degradation before reaching the 0.05 rad cycle due to localized bolted splice failure. This indicates that for deep beam sections, a skew angle threshold of 20° should be observed to prevent premature splice-induced failure in seismic applications.
2. Energy Dissipation Mechanism: Energy dissipation in column-tree systems is governed by a combination of splice friction and inelastic deformation. As the skew angle increased to 30°, the total dissipated energy at 0.04 rad decreased by 2.9–8.9%. The inelastic components were more sensitive to the skew, showing a reduction of 5.4–15.4%, whereas the frictional component decreased by only 1.7–5.4% compared to orthogonal models.
3. Weld Fracture Susceptibility: Out-of-plane skew disrupted the symmetry of fracture indices (*TR*, *PI*, and *RI*) along the beam flange, shifting peak demands toward the medial joint. Nevertheless, these peak values increased only modestly, suggesting that skew angles up to 30° do not critically exacerbate the fracture risk of the welded beam-to-column interface.
4. Splice Bolt Performance: While significant pretension loss occurred as the drift increased, the skew angle had a minimal impact on the bolt-force history up to 0.04 rad. At this drift level, flange and web bolt forces decreased to 0.39–0.50*T_o_* and 0.41–0.64*T_o_*, respectively. Localized splice deterioration in deep beam configurations was primarily confined to the extreme 0.05 rad cycles.
5. Deformation Components: Splice rotation was a dominant contributor to system flexibility, accounting for 45–65% of the total story drift, depending on beam depth. Interestingly, the introduction of skew led to a slight reduction in splice rotation levels at 0.04 rad in most configurations.
6. Column twisting: Although column twisting increased linearly with the skew angle, it remained below 0.5° even at a 30° skew. This indicates that out-of-plane skew has a limited impact on the global stability of the column member within the investigated range.

In conclusion, column-tree moment connections with an out-of-plane skew up to 30° provide sufficient seismic performance for SMF applications. These findings support the implementation of skewed connections in complex building geometries without compromising structural integrity. However, to prevent unexpected splice failure at high drift levels, it is recommended to limit the skew angle to 20° for deep beam applications.

## Figures and Tables

**Table 1:** Splice connection components.

Model No.	Beam Size	Flange Splice		Web Splice	
		Plates	Bolts	Plates	Bolts
4	H-400 × 200 × 8 × 13	External PL-10 × 410 × 200	12-M20	2PL-6 × 310 × 210	8-M20
		Internal 2PL-12 × 410 × 75			
5	H-500 × 200 × 10 × 16	External PL-14 × 530 × 200	16-M20	2PL-6 × 380 × 210	10-M20
		Internal 2PL-14 × 530 × 75			
6	H-600 × 200 × 11 × 17	External PL-12 × 530 × 200	16-M20	2PL-9 × 450 × 210	12-M20
		Internal 2PL-12 × 530 × 75			

**Table 2:** Performance comparison between analysis results and test data.

Model No.	Result	Initial Stiffness, *K_i_*	Average Slip Load During 0.02 rad Cycles, *P_s_*	Maximum Load at 0.05 rad, *P_u_*
		[kN/mm]	[kN]	[kN]
4	Test [4]	2.16	35.0	109.3
	FEA	2.15 (error: −0.5%)	37.3 (error: +6.6%)	116.4 (error: +6.5%)
5	Test [4]	3.57	91.1	217.8
	FEA	3.84 (error: +7.5%)	96.2 (error: +5.6%)	224.8 (error: +3.2%)
6	Test [4]	3.95	101.1	278.7
	FEA	3.67 (error: −7.1%)	103.9 (error: +2.8%)	272.9 (error: −2.1%)

**Table 3:** Energy dissipation component up to the end of 0.04 rad.

Model No.	Skew Angle	Cumulative Energy Dissipation		
		Inelastic [kJ]	Friction [kJ]	Total [kJ]
4	No Skew	37.3	78.2	115.5
	10°	37.0 (−0.80%)	78.0 (−0.26%)	115.0 (−0.43%)
	20°	36.3 (−2.68%)	77.5 (−0.90%)	113.8 (−1.47%)
	30°	35.3 (−5.36%)	76.9 (−1.66%)	112.2 (−2.86%)
5	No Skew	92.8	120.7	213.5
	10°	91.6 (−1.29%)	120.0 (−0.58%)	211.6 (−0.89%)
	20°	87.7 (−5.50%)	117.6 (−2.57%)	205.3 (−3.84%)
	30°	84.4 (−9.05%)	114.2 (−5.39%)	198.6 (−6.98%)
6	No Skew	145.2	144	289.2
	10°	141.7 (−2.41%)	143.9 (−0.07%)	285.6 (−1.24%)
	20°	128.8 (−11.29%)	142.1 (−1.32%)	270.9 (−6.33%)
	30°	122.8 (−15.43%)	140.6 (−2.36%)	263.4 (−8.92%)

Note: The value in parentheses indicates the percentage difference compared to the base model with no skew.

## Data Availability

The original contributions presented in the study are included in the article. Further inquiries can be directed to the corresponding author.

## References

[1] Cucuzza R., Aloisio A., Rad M.M., Domaneschi M. (2024). Constructability-Based Design Approach for Steel Structures: From Truss Beams to Real-World Inspired Industrial Buildings. Autom. Constr..

[2] (2017). Seismic Design Code for Steel Structures.

[3] (2022). Seismic Provisions for Structural Steel Buildings.

[4] Oh K.Y. (2019). Seismic Performance Evaluation of Column-tree Steel Moment Connections. Ph.D. Thesis.

[5] (2019). Structural Design Code for Steel Buildings.

[6] (2016). Specification for Structural Steel Buildings.

[7] Karami B., Hashemi B.H., Ramhormozian S., Zarabimanesh Y. (2022). Numerical Study on Prequalification and Cyclic Performance of Column-tree Connections. Structures.

[8] Hamburger R.O., Krawinkler H., Malley J.O., Adan S.M. (2009). NEHRP Seismic Design Technical Brief No. 2, Seismic Design of Steel Special Moment Frames: A Guide for Practicing Engineers.

[9] Kim D.W., Sim H.B., Uang C.M. (2010). Cyclic Testing of Non-orthogonal Steel Moment Connections for LAX TBIT Modification.

[10] Kim D.W., Hall S.C., Sim H.B., Uang C.M. (2016). Evaluation of Sloped RBS Moment Connections. J. Struct. Eng..

[11] Hong J.K. (2019). Sloped RBS Moment Connections at Roof Floor Subjected to Cyclic Loading: Analytical Investigation. Int. J. Steel Struct..

[12] Prinz G.S., Richards P.W. (2016). Demands on Reduced Beam Section Connection with Out-of-Plane Skew. J. Struct. Eng..

[13] Desrochers C., Prinz G.S., Richards P.W. (2018). Column Axial Load Effects on the Performance of Skewed SMF RBS Connections. J. Constr. Steel Res..

[14] Asl M.H., Saeidzadeh M., Momenzadeh S. (2019). Evaluation of Friction Strength Loss in Endplate Moment Connections with Skewed Beam. Int. J. Steel Struct..

[15] Hong J.K. (2023). Analytical Study on Performance of Column-tree Steel Moment Connections with Out-of-plane Skewed Beam. J. Arch. Inst. Korea.

[16] ABAQUS (2022). ABAQUS/CAE Users’ Guide.

[17] Verein Deutscher Ingenieure (2014). VDI 2230 Part 2: Systematic Calculation of Bolted Joints—Multi-Bolted Joints.

[18] Grazejd R. (2025). Modelling of the Bolted Joint in Relation to the Working Load of the Bolt. Wseas Trans. Appl. Theor. Mech..

[19] Matthews P., Nunez E. (2023). Cyclic Behavior of the Column-Tree Moment Connection with Weakened Plates: A Numerical Approach. Buildings.

[20] Kaufmann E.J., Metrovich B.R., Pense A.W. (2001). Characterization of Cyclic Inelastic Strain Behavior on Properties of A572 Gr. 50 and A913 Gr. 50 Rolled Sections.

[21] Hartioper A.R., Sousa A.C., Lignos D.G. (2021). Constitutive Modeling of Structural Steels: Nonlinear Isotropic/Kinematic Hardening Material Model and Its Calibration. J. Struct. Eng..

[22] Cho E.S., Hyun J.H., Han S.W. Estimation of Combined Hardening Model Parameter Values for Korean Steel Grades. Proceedings of the 2020 World Congress on Advances in Civil, Environmental, & Material Research (ACEM20).

[23] Xu J., Wang Z., Wang P., Pan B., Li B. (2021). Numerical Investigations on Large Size Stiffened Angle Connections with Different Bolt Patters. J. Constr. Steel Res..

[24] El-Tawil S., Mikesell T., Kunnath S.K. (2000). Effect of Local Details and Yield Ratio on Behavior of FR Steel Connections. J. Struct. Eng..

